# Non-small Cell Lung Cancer With Proto-Oncogene B-Raf V600E Presenting a Distinctive Clinical Course: A Case Report

**DOI:** 10.7759/cureus.23055

**Published:** 2022-03-11

**Authors:** Aosa Sasada, Tatsuya Yuba, Shinsuke Shiotsu, Taisuke Tsuji, Noriya Hiraoka

**Affiliations:** 1 Respiratory Medicine, Kyoto Daiichi Red Cross Hospital, Kyoto, JPN; 2 Clinical Oncology, Kyoto Daiichi Red Cross Hospital, Kyoto, JPN

**Keywords:** long-term survival, lung adenocarcinoma, lung cancer, case report, braf v600e mutation

## Abstract

Cases of proto-oncogene B-Raf (*BRAF*) V600E mutation are rare, accounting for 1%-4% of non-small cell lung cancers (NSCLCs), and its clinical features remain unclear. Here, we report a case of* BRAF* mutation-positive lung adenocarcinoma with an atypical clinical course and long-term survival. The patient was a 63-year-old female nonsmoker who was diagnosed with stage IA adenocarcinoma after surgical resection. Five years after the surgery, cancer recurred and was treated with various cytotoxic anticancer agents. During the course of treatment, the patient was found to be *BRAF *V600E mutation-positive and was treated with molecular-targeted drugs. Although multiple brain, subcutaneous, and tonsillar metastases appeared, the progression was significantly slower, and the patient survived for 14 years and three months after the diagnosis. There have been few case reports of long-term survival in *BRAF*-positive lung cancer, and more cases need to be accumulated in the future to gather more information. Based on this case, we speculate that sensitivity to cytotoxic anticancer agents such as pemetrexed (PEM) and maintenance of performance status (PS), in addition to molecular-targeted agents, are important for long-term survival.

## Introduction

It has been reported that 90% of lung cancers are non-small cell lung cancer (NSCLC), and 3.5%-5% of NSCLC cases have B-Raf proto-oncogene (BRAF) mutations. V600E corresponds to half of the BRAF mutated cases [1]. Many mutations and rearrangements associated with NSCLC, such as those in epidermal growth factor receptor (EGFR), anaplastic lymphoma kinase (ALK), and c-ros oncogene 1 (ROS1), have been recently revealed. Some of them present with unique clinical features, although there are only a few reports of the clinical features of NSCLCs with BRAF mutations.

We present a case of a female patient, a nonsmoker, diagnosed with stage IA adenocarcinoma of the lung, initially treated with surgery, who experienced recurrence with distant metastasis involving the brain in a short period of time and was resistant to several chemotherapies. EGFR, ALK, and ROS1 mutations were not identified in the tumor cells of this patient. While she was undergoing chemotherapy, it was revealed that the tumor cells had a BRAF V600E mutation. Combination therapy with dabrafenib and trametinib resulted in progression-free survival for approximately 10 months. Twenty months after starting the treatment, chemotherapy was terminated, and she died one month later. She had survived for 14 years and three months after surgery and 10 years and two months after the recurrence. Her clinical course was different from the typical course of lung adenocarcinomas and, thus, was worth reporting.

## Case presentation

**Figure 1:**
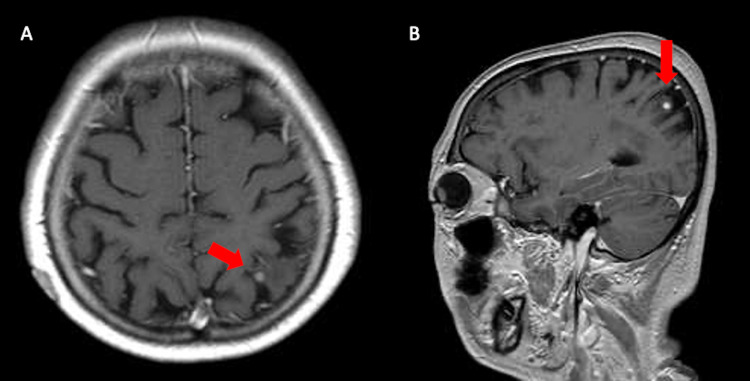
MRI of the head two years after the recurrence. These are (A) horizontal and (B) sagittal sections of a gadolinium contrast-enhanced T1-weighted MRI performed for screening. A 4 mm strongly contrasted nodule (red arrow) was seen in the left parietal lobe with surrounding edema. There were no other lesions suspicious for metastasis.

**Figure 2:**
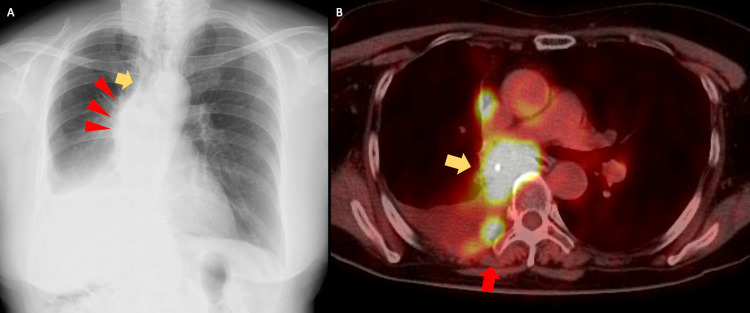
(A) Chest X-ray at the time of the initial examination. (B) PET-CT image at the time of the initial examination. (A) Chest X-ray showed thickening of the right paratracheal line (yellow arrow) and protrusion of the right first arch (red arrowhead) and decreased permeability of the right lower lung field due to pleural effusion. (B) PET-CT revealed a mass near the end of the right lower lobe bronchus postoperatively (yellow arrow), with strong 18F-labeled fluorodeoxyglucose accumulation in the same area. In addition, right pleural dissemination, eighth and ninth rib metastasis (red arrow), and multiple mediastinal lymph node metastasis were observed. PET-CT, positron emission tomography-computed tomography

The patient was a 63-year-old female with a past medical history of hypertension and dyslipidemia, without any history of smoking or drinking. She visited the previous hospital because an abnormality, which was suspected to be lung cancer, was found on her chest radiograph. She underwent surgery in 2005 and was diagnosed with stage IA pT1N0M0 lung adenocarcinoma, as per her preoperative diagnosis. Five years after the initial diagnosis, an endobronchial tumor near the postoperative margin and heterogeneous thickening of the pleura appeared, and bronchoscopic tumor biopsy and pleural biopsy with video-assisted thoracic surgery detected an adenocarcinoma, which was determined to be a recurrence. No EGFR or ALK mutation was detected. Although the details of this treatment are unclear because it was administered by her previous doctor, she received first-line chemotherapy with carboplatin and paclitaxel for four cycles and focal radiation therapy to the recurrent intratracheal tumor (30 Gy/10 fractions) and one pleural nodule (30 Gy/10 fractions). Second-line therapy, comprising a combination of cisplatin and docetaxel for four cycles, was administered to the patient, although she had progressive disease. After that, she was treated with tegafur/gimeracil/oteracil (S-1), although it was ineffective. Two years after recurrence, brain metastasis was found (Figure 1). At this point, her performance status (PS) was zero, and she had no neurological symptoms. Gamma-knife treatment was started. Subsequent treatment was performed at our hospital at the patient’s request. The images taken at her first visit to our hospital are presented in Figure 2.

**Figure 3:**
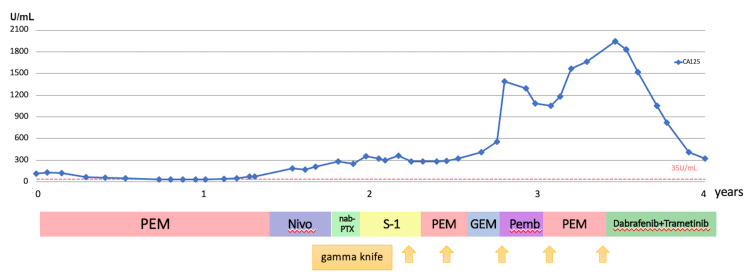
Clinical course of CA125 and therapy. CA125 reflected tumor aggressiveness; it was kept low when PEM was first used, then increased with disease progression, and decreased again with dabrafenib and trametinib treatment. CA125, cancer antigen 125; PEM, pemetrexed; Nivo, nivolumab + pembrolizumab; Nab-PTX, nanoparticle albumin-bound paclitaxel; S-1: tegafur/gimeracil/oteracil; GEM, gemcitabine; Pemb, pembrolizumab

**Figure 4:**
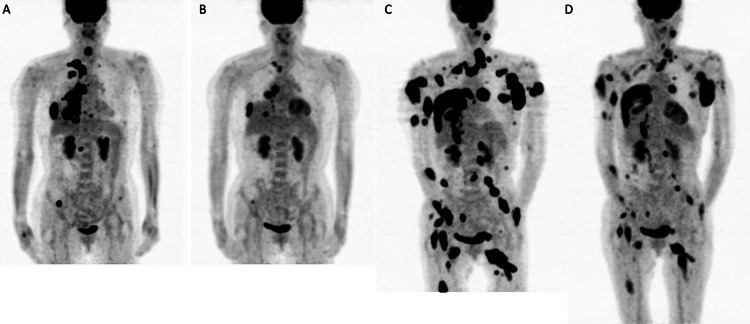
Changes in PET-CT findings during treatment. (A) PET-CT image at the time of the initial examination. (B) PET-CT image after 10 months of pemetrexed treatment. The local recurrent mass had shrunk, and the accumulations in this area, pleura, and iliac crest were all reduced. (C) PET-CT image before molecular-targeted drug treatment. 18F-Labeled fluorodeoxyglucose accumulation was observed in soft tissues, such as muscle and subcutaneous tissues, and in the left palatine tonsil. (D) PET-CT image six months after the start of molecular-targeted drug therapy. Various accumulations were reduced, suggesting that treatment was effective. PET-CT, positron emission tomography-computed tomography

Carcinoembryonic antigen and cytokeratin 19 fragment are known tumor markers for NSCLC. They did not show a significant increase in previous medical examinations. While cancer antigen 125 (CA125) is commonly known as a gynecological tumor marker for ovarian and uterine cancers, there are also reports that it may be a prognostic factor for NSCLC [2,3]. We searched for other tumor markers and found that CA125 was related to disease activity. She received pemetrexed (PEM), which is one of the drugs used in the first-line regimen for non-small and non-squamous cell lung cancer, which improved the clinical symptoms and decreased the tumor size; CA125 levels also decreased (Figure 3). Positron emission tomography/computed tomography findings showed a marked improvement (Figure 4B).

**Figure 5:**
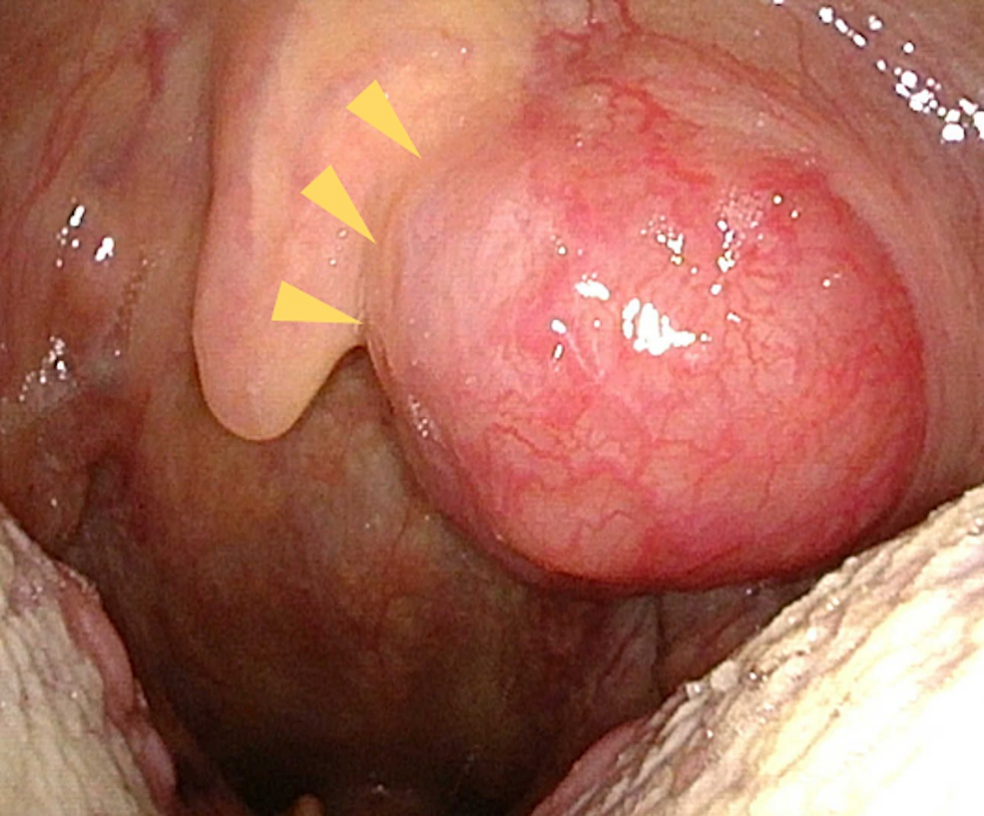
Metastatic lesion to the palatine tonsil. A mass was found on the left palatine tonsil (yellow arrow) revealing metastasis of lung adenocarcinoma based on biopsy results.

We determined that PEM monotherapy was indeed effective but failed after 21 cycles. Although the patient received various chemotherapy regimens after that, including the administration of immune checkpoint inhibitors (nivolumab and pembrolizumab) and readministration of previous drugs, the disease progressed. Multiple metastatic lesions to atypical organs, such as subcutaneous tissue and the left tonsil (Figure 5), were found. Due to the frequent occurrence of minute brain metastases, gamma-knife treatment was repeated. The BRAF V600E mutation was detected as a result of comprehensive genetic analysis by next-generation sequencing, 11 years and three months after the onset. At that time, dabrafenib and trametinib, which were considered to be effective against cancers with this mutation, were not approved in Japan. Thus, we continued the other chemotherapies (Figure 3). The patient started using these two drugs as soon as the approval was provided, 12 years and seven months after the onset. This treatment improved the clinical symptoms and tumor size and decreased the CA125 level again with no adverse events (Figures 3, 4). However, approximately 10 months after starting dabrafenib and trametinib combination therapy, CA125 levels increased gradually. Palpable metastatic skin lesions also increased in number. We decided that this was caused by the progression of the disease. Because there was no effective alternative agent and the patient wanted to continue the treatment, we continued administering dabrafenib and trametinib. Twenty months after starting the combination therapy, we terminated the therapy as the patient experienced difficulty in taking the medicine. One month later, the patient died.

## Discussion

BRAF mutation is a rare mutation occurring in 1%-4% [4-7] of NSCLCs, and the understanding of its natural history remains unclear. Regarding prognosis, Marchetti et al. reported that compared to BRAF mutation-negative NSCLC, patients with the BRAF V600E mutation had a poor prognosis with disease-free survival (DFS) of 15.2 months versus 52.1 months and overall survival (OS) of 29.3 months versus 72.4 months, respectively [1]. The patient survived for 172 months from disease onset and 122 months after recurrence. The median OS of BRAF mutation-positive lung cancer with stage IV or metastatic recurrence ranges from 15.2 months [4] to 28.1 months [8]. This case clearly shows better long-term survival compared to previous reports.

Molecular-targeted drugs, dabrafenib and trametinib combination therapy, are clinically effective in BRAF mutation-positive lung cancer [1]. However, their tumor-suppressive effect is limited (PFS, 10.9 months; OS, 24.6 months) [9], which does not explain the long-term survival of this patient.

Previous studies have shown that a certain number of BRAF-positive lung cancers are slow-growing and patients survive for more than five years after the detection of distant metastasis, regardless of the use of molecular-targeted drugs [7,10,11]. These groups are treated with various therapies, such as cytotoxic anticancer drugs and immune checkpoint inhibitors, in addition to molecular-targeted drugs. In this case, the tumor proportion score (TPS) of the programmed death-ligand 1 measured in the skin metastases was 70%, which was high. Thus, the patient was treated with nivolumab and pembrolizumab, but both treatments were only effective for a short time (three months). Dudnik et al. reported that the effect of immune checkpoint inhibitors on BRAF mutation-positive lung cancer did not correlate with the programmed death-ligand 1 expression rate [12], which is consistent with their report.

On the other hand, this patient responded well to cytotoxic anticancer agents, such as PEM. There have been several case reports of successful treatment of BRAF-positive lung cancer with PEM [7,10]. In a study by Paik et al., PEM was included in the regimens that were effective in treating patients with stage IIIB disease or those with stable disease [7]. Generally, the expression of thyroid transcription factor-1 (TTF-1) is involved in the response to PEM. In this case, as well, the patient was TTF-1-positive. In addition, patients with a low expression of thymidylate synthase (TS) respond well to PEM, which may be useful as a biomarker for predicting efficacy [13], although this was not studied in this case.

Myall et al. reported that performance status (PS) was an independent prognostic factor in case reports of long-term survivors compared with short-term survivors, with no significant differences in the patient background such as age or sex [11]. The patient had a very good PS, maintaining PS1 until one month before her death. The correlation between the presence or absence of the V600E mutation and prognosis varies across reports. In BRAF mutation-positive lung cancer, molecular and clinical heterogeneities have been pointed out [11].

The clinical course of the patient in this case report was significantly diverse, with numerous metastases in atypical sites for lung cancer, such as the skin, intramuscular regions, and palatine tonsils. Skin metastasis is generally associated with poor prognosis and is usually observed after the disease has progressed and metastasized to other parts of the body. However, there have been reports of this being observed in BRAF mutation-positive lung cancer from the time of initial diagnosis [14]. Therefore, in the case of BRAF-positive lung cancer, skin metastasis would not necessarily be a poor prognostic factor.

In this case, brain metastasis occurred 28 months after recurrence. Gamma-knife therapy was administered, followed by periodic head MRI. Six gamma-knife treatments at 106 sites were performed to prevent PS decline due to brain metastasis. In cases where long-term survival has been achieved, even if distant metastasis has occurred, the progression is slow, and long-term survival may be expected by maintaining PS and continuing treatment with local therapy, such as irradiation.

Our results suggest that in the treatment of BRAF-positive lung cancer, sensitivity to molecular-targeted drugs and cytotoxic anticancer agents, including PEM, and the maintenance of PS may lead to long-term survival. For the selection of cytotoxic anticancer agents, it would be desirable to investigate TTF-1 and TS expression at the time of diagnosis.

## Conclusions

In conclusion, we report a case of a patient with BRAF mutation-positive NSCLC with distant metastasis who achieved long-term survival. Although further accumulation of cases is necessary, the present results suggest that in the treatment of BRAF-positive lung cancer, sensitivity to cytotoxic anticancer and molecular-targeted agents and maintenance of PS may lead to long-term survival.
